# Evaluation of anaplastic thyroid carcinoma in the Kurdistan region of Iraq

**DOI:** 10.1186/s12893-022-01810-w

**Published:** 2022-10-21

**Authors:** Dilshad Hamad Mustafa, Baderkhan Saeed Ahmed, Rawand Musheer Haweizy, Azhy Muhammed Dewana

**Affiliations:** 1 Department of Faciomaxillary Surgery, College of Dentistry, Hawler Medical University, Erbil, Iraq; 2 Department of Surgery, College of Medicine, Hawler Medical University, Erbil, Iraq

**Keywords:** Anaplastic thyroid carcinoma, Surgery, Multimodal therapy, Prognosis, Survival, Kurdistan region Iraq

## Abstract

**Background:**

Anaplastic thyroid carcinoma is a rare and lethal disease that accounts for 1–2% of thyroid malignancies. It is an aggressive locoregional disease with a high rate of distant metastasis, a poor prognosis, and a mean survival rate of 3–6 months after diagnosis. This retrospective study aimed to analyse the clinical and pathological features of ATC to assess treatment procedures and its outcome.

**Methods:**

We analysed data from 22 patients diagnosed with ATC from 2018 to 2021, using the Kaplan-Meier method and log-rank test to determine overall survival.

**Results:**

Patients’ median age was 64.3 ± 17.1 years. Females were more affected (male/female ratio: 1:1.7); 14 cases occurred in females (63.6.4%), and eight in males (36.4%). The most common manifestations were neck enlargement (81.8%) and dyspnoea (72.27%), and the tumour size was > 4 cm in 17 (77.3%) patients. The percentage of cases that presented in clinical-stage IVA was 36.4%, with 31.8% presenting in clinical-stage IVB and 31.8% presenting in clinical-stage VIB. Among 22 cases, 14 (63.6%) were operable, and 8 (36.4) were inoperable (p = 0.015). Multimodal therapies were associated with better survival (surgery plus radiotherapy without systemic treatment, *P* = 0.063). The median overall survival was three months (IC 95%, 0.078–5.922). One-year and two-year survival rates were 9% and 4.5%, respectively.

**Conclusion:**

ATC is a rapidly growing cancer that, fortunately, is rare. Early diagnosis and multimodality treatment may provide a better quality of life and survival time for this group of patients.

**Supplementary Information:**

The online version contains supplementary material available at 10.1186/s12893-022-01810-w.

## Background

Anaplastic thyroid carcinoma (ATC) is an uncommon but profoundly aggressive thyroid malignancy established in undifferentiated follicular thyroid cells [1, 2]. It accounts for more than 50% of all deaths attributable to thyroid tumours, with a mean survival period of three to four months and disease-specific mortality at one year of about 100% [1, 3]. ATC prevalence varies from one place to another, with higher rates in goitre-endemic areas; in the USA it accounts for 1.7% of all thyroid malignancies, ranging from 1.3 to 9.8% [4]. It is commonly diagnosed in elderly persons (the mean age at diagnosis is 65–70 years), with a slightly higher female prevalence [5]. The yearly frequency of ATC is roughly one-to-two cases per million people.

Risk factors for the development of ATC include a history of radiation to the neck or chest, long-standing goitre, progressed age, and a history of benign or malignant thyroid disease [6]. Patients with ATC encounter noteworthy nearby compressive symptoms due to a quickly advancing central neck mass (77%), accompanying dysphagia (40%), hoarseness (40%), and stridor (24%). Metastases are well known in 50% of patients at the time of diagnosis, most commonly in the lungs (80%), bone (6–16%), and brain (5–13%) [7, 8].

The diagnostic modality for ATC is fine needle aspiration cytology (FNAC). In doubtful cases, the diagnosis is made by histology on a tissue biopsy [9]. Because of its aggressive behaviour and low chance of recovery, ATC is classified according to the American Joint Committee on Cancer TNM (AJCC-8) system as T4 and stage IV [10, 11].

Contrast-enhanced neck computed tomography (CT) is recommended to assess the advancement of tumour attack and lymph node metastasis. For the assessment of far-off metastases, fluorodeoxyglucose (FDG), positron emission tomography (PET) and brain magnetic resonance imaging (MRI) are commonly prescribed [12].

Multidisciplinary healthcare teams (comprising surgeons, radiation oncologists, medical oncologists, psychologists, and nurses, among others) are inherently involved in ATC treatment, and interdisciplinary communication and collaborative working can represent a significant challenge during ATC management. A significant number of ATC patients are non-operable at the time of diagnosis of their cancer. Within the subset of patients with operable disease at the time of diagnosis, surgery combined with postoperative radiation +/- chemotherapy may be useful [1, 13].

The purpose of this study is to retrospectively analyse clinical and pathological features of ATC to assess treatment procedures and outcomes.

## Methods

### Patients and methods

In this retrospective study, 700 cases of thyroid cancer were examined from the 1st of June 2018 to the 31st of December 2021). Of these 700 cases, 22 were included as cases of ATC. The data were obtained from various hospitals and health care centres (Rizgary, Nanakaky, Hewa, and Azadi Teaching Hospitals, with their oncologic units) throughout the main cities of the Kurdistan Region (Erbil, Sulaymaniyah, and Duhok) in northern Iraq. All 22 cases were pathologically diagnosed as ATC, depending on FNAC and excisional biopsy.

Preoperative investigation documentation such as that concerning laryngoscopy, neck ultrasonography, and CT of the head, neck, chest, and abdomen were examined to detect the extent of tumour invasion. Two conditions comprised exclusion criteria: poorly differentiated thyroid cancer and patients’ refusal to participate. A questionnaire was administered to record participants’ demographic characteristics (age, sex, place of residence) and their clinical presentation, diagnostic procedures, therapeutic approaches and outcomes, and survival. Additional data was collected from patients’ relatives by direct interviewing or calling.

Prognostic factors like survival rates by age, tumour size, presence of distant metastasis, and treatment were calculated. The guidelines of the American Joint Committee on Cancer were used for staging: tumour (T), nodal (N), and distant metastasis (M).

This study is part of an ongoing Ph.D. research project sponsored by the College of Medicine at Hawler Medical University. It was approved by the Ethical Committee in the College of Medicine (meeting code: 6, paper code: 6, date: 27/04/2022).

### Statistical analysis

Data were analysed using the SPSS version 25. Numerical variables were summarised by calculating the means and the standard deviations (SDs). Categorical variables were presented in the form of frequencies and proportions. Kaplan-Meier survival analysis was applied, and Log Rank (Mantel-Cox) test was used to compare the survival curves of the studied variables (like age, gender, grading, and size). A *P* value of ≤ 0.05 was considered statistically significant.

## Results

A total of 22 patients agreed to participate in this study, comprising 14 females (63.6%) and eight males (36.4%). The male-to-female ratio was 1:1.7. The mean age was 64.3 ± 17.1 years, ranging from 26 to 87 years. The majority (n = 14, 63.6%) were aged ≥ 60 years.

The most common clinical presentation was swelling in the anterior neck’s lower part, which was observed in 18 (81.8%) patients. It was bilateral in a third of them (n = 6, 33.3%). More than a third (n = 8, 36.4%) of patients were stage A, and the tumour size was > 4 cm in 17 (77.3%) patients.

The majority (n = 20, 90.9%) of patients were diagnosed by FNAC, and the rest (n = 2, 9.1%) by excisional biopsy as ATC. The ATC-related clinical characteristics of participants are shown in Table 1.

**Table 1:** Characteristics of patients with ATC

	No.	(%)	Mean (SD)	Median	Min.	Max.
**Gender**						
Male	8	(36.4)				
Female	14	(63.6)				
**Age at diagnosis**			64.3 (17.1)	68.0	26.0	87.0
< 60 years	8	36.4				
≥ 60 years	14	63.6				
**Clinical presentation**						
Lump	18	(81.8)				
Pain	3	(13.6)				
Dyspnoea	16	(72.7)				
Dysphagia	10	(45.5)				
Hoarseness of voice	9	(40.9)				
Stridor	2	(9.1)				
**Site of lump**	18					
Right	4	(22.2)				
Left	4	(22.2)				
Bilateral	6	(33.3)				
Diffuse	4	(22.2)				
**Staging** **Stage 4**						
A. Local infiltration	8	(36.4)				
B. Regional involvement of lymph nodes	7	(31.8)				
C. Distant metastasis	7	(31.8)				
**Tumour size**			5.8 (2.0)	5.7	3	12
≤ 4 cm	5	(22.7)				
> 4 cm	17	(77.3)				
**WBC**			8911 (4354)	8350	2800	18,000
**Fine needle aspiration**						
Indeterminate*	2	(9.1)				
Malignant (anaplastic)	20	(90.9)				
Total	22	(100.0)				

*Proved post-operatively by tissue biopsy to be malignant

Different methods were used for the treatment of the 22 included cases, of which 14 (63.6%) were operable. Six patients (27.3% of total cases) underwent total thyroidectomy with neck dissection, while the rest had other techniques of surgery like lobectomy (4 cases), subtotal thyroidectomy (1 case) and debulking (3 cases). The multimodal method was applied only for two cases. Supportive interventions such as tracheostomy were undertaken for 3 (13.6% of total cases) patients due to airway obstruction, and one (4.5%) patient had percutaneous endoscopic gastrostomy. Participants’ therapeutic modalities are described in Table 2.

**Table 2:** Treatment modalities

	No.	%)
Type of surgery		
Lobectomy	4	(18.2)
Subtotal thyroidectomy	1	(4.5)
Total thyroidectomy	6	(27.3)
Debulking	3	(13.6)
Inoperable	8	(36.4)
Type of neck dissection (n = 6)		
Central	3	(50.0)
Central and lateral	3	(50.0)
Type of therapy		
Chemotherapy	2	(9.1)
Radiotherapy	1	(4.5)
Palliative Surgery*	4	(18.2)

*Surgery: - supportive interventions such as tracheostomy were undertaken for three patients, and one patient had percutaneous endoscopic gastrostomy as palliative therapy for those inoperable cases

The mean survival rate was 5.5 months, and the median was 3.0 months. Two (9%) patients who got thyroidectomy, chemotherapy and radiotherapy lived longer than one year, of whom only one case (4.5%) was alive without any evidence of recurrence of the disease at 40-month follow-up. There was no significant association between survival rate in relation to age (*P* = 0.805) and gender (*P* = 0.103) (Table 3).

**Table 3:** Survival by age and gender

	Mean				Median				
			**95% Confidence Interval**				**95% Confidence Interval**		P*
	**Estimate**	**SE**	**Lower Bound**	**Upper Bound**	**Estimate**	**SE**	**Lower Bound**	**Upper Bound**	
Age (y)									
< 60	5.16	1.74	1.75	8.57	4.00	3.67	0.00	11.20	
≥ 60	5.71	1.54	2.68	8.74	3.00	0.92	1.18	4.81	0.805
Gender									
Male	3.16	1.24	0.71	5.61	1.00				
Female	6.57	1.54	3.55	9.58	4.00	1.87	0.33	7.66	0.103
Overall									
	5.55	1.17	3.24	7.85	3.00	1.49	0.07	5.92	

*By Log Rank (Mantel-Cox)

**Fig. 1:**
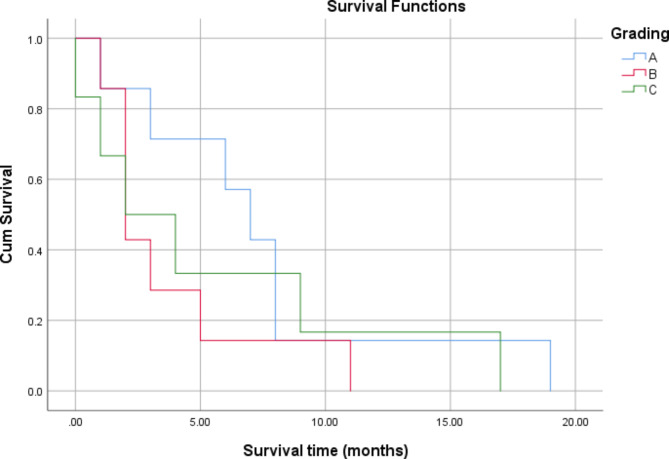
Survival by grading

**Fig. 2:**
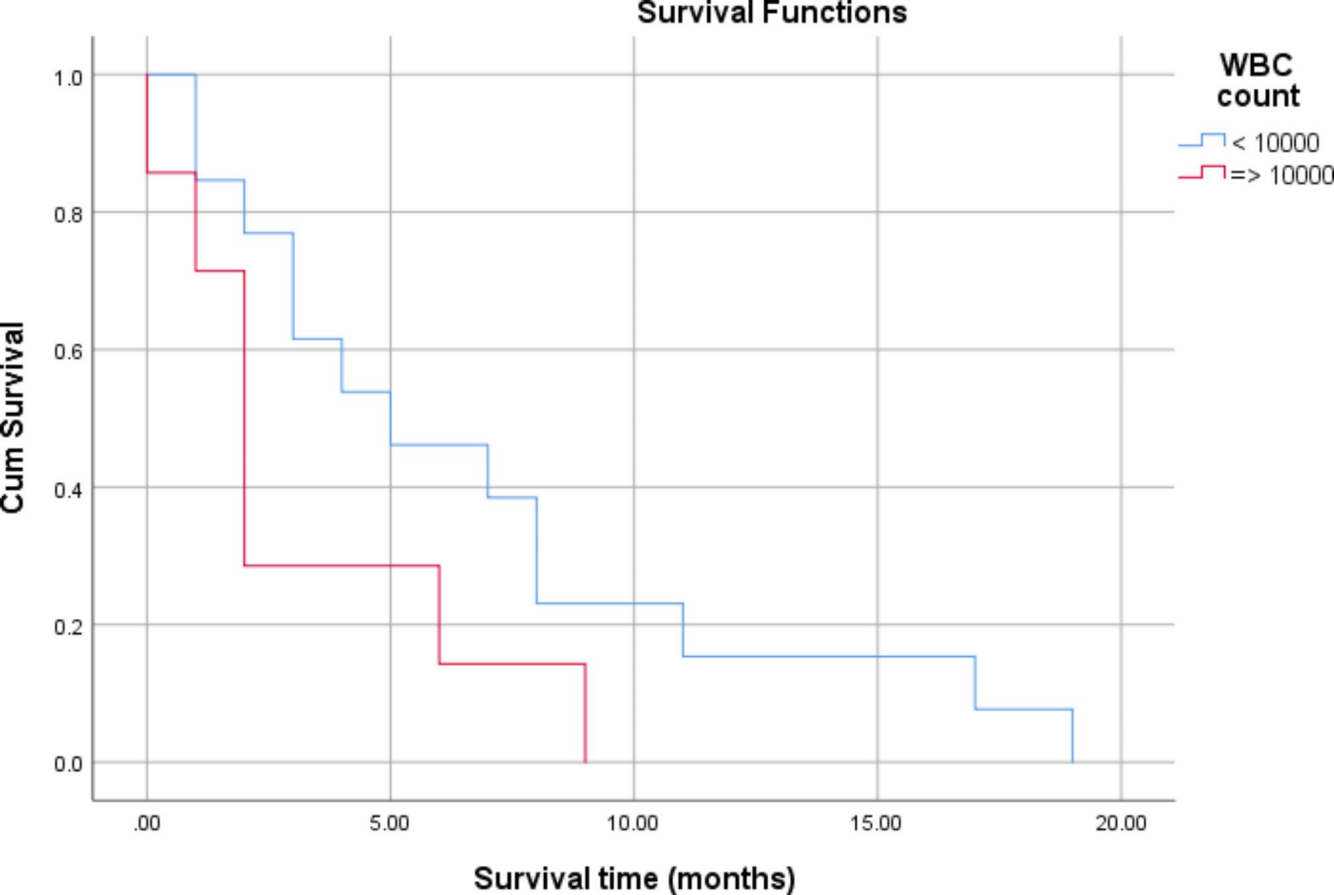
Survival by WBC count

**Fig. 3:**
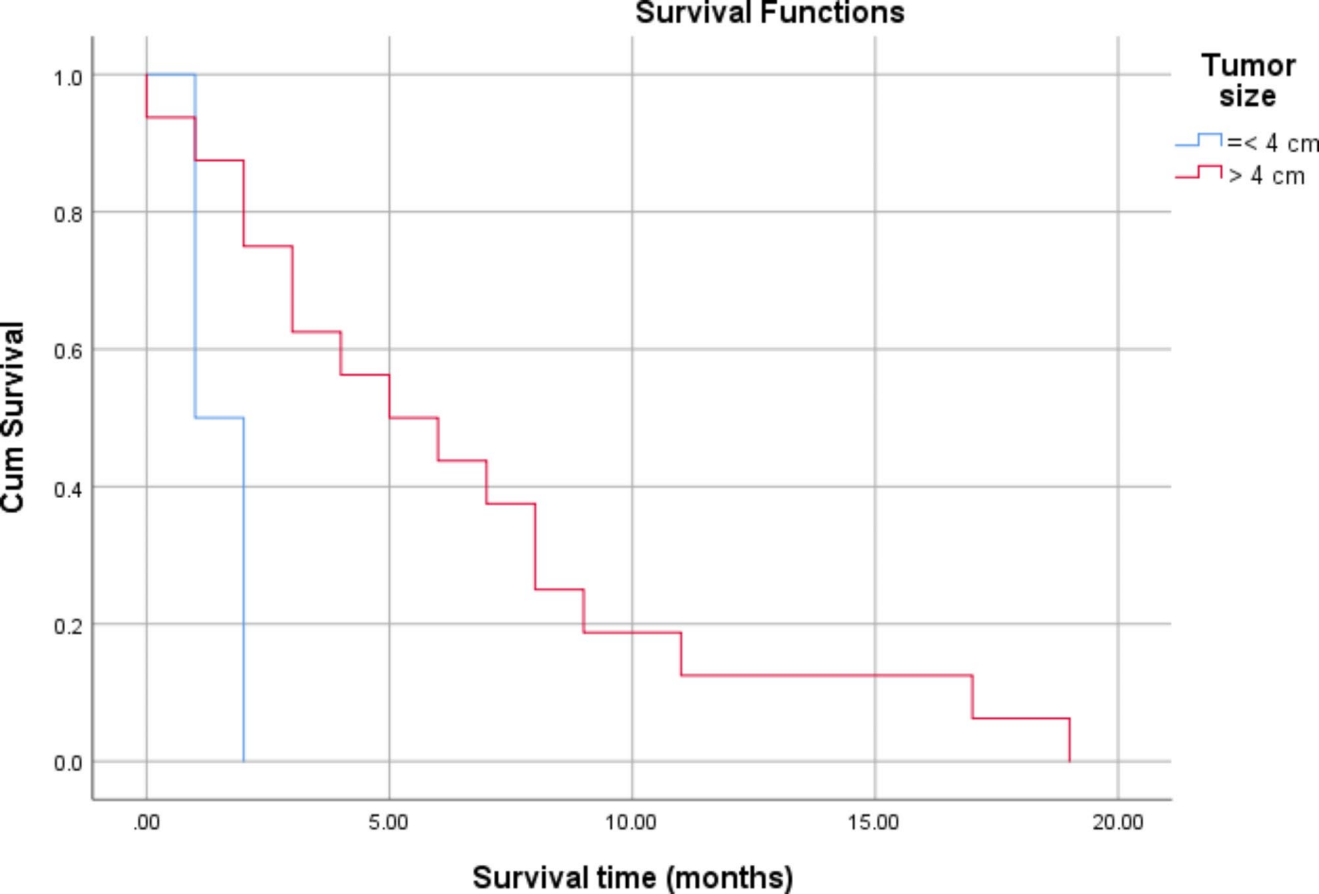
Survival by tumor size

**Fig. 4:**
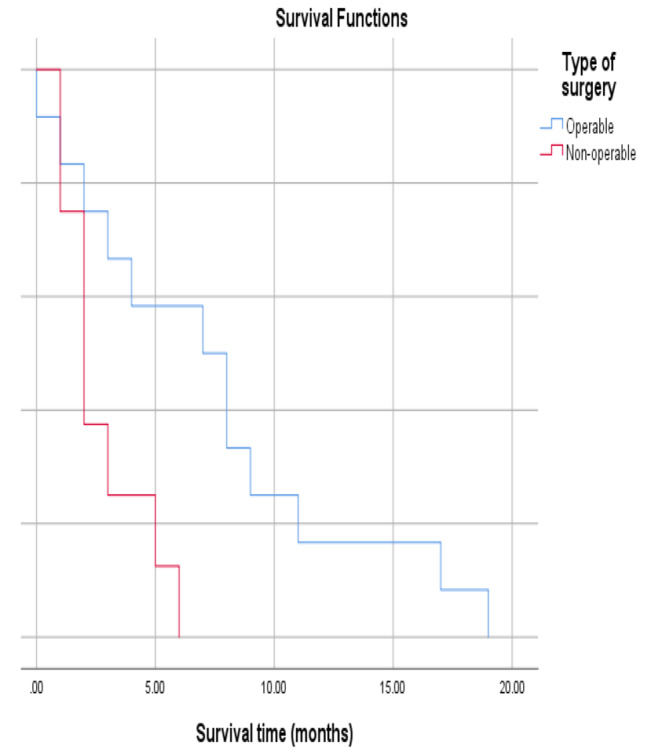
Survival by intervention

**Fig. 5:**
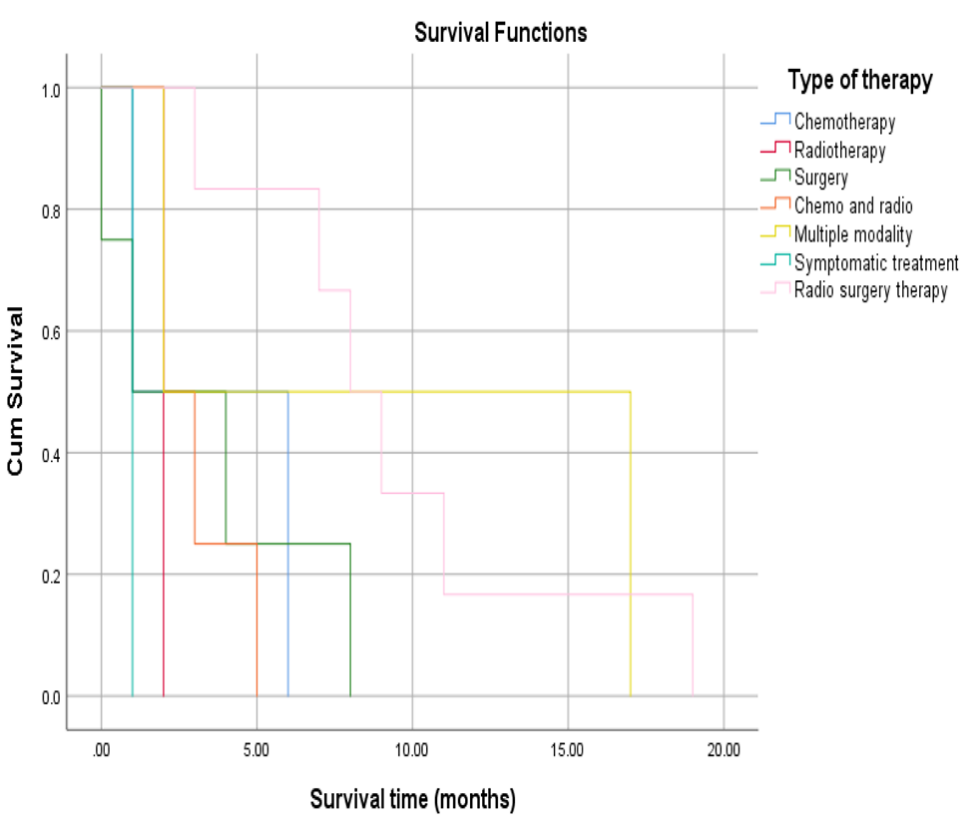
Survival by type of therapy

There was no significant association between the survival of patients with tumour grade (*P* = 0.441) and White blood cell (WBC) count (*P* = 0.104), as shown in Table 4; Figs. 1 and 2. Significant associations were found between tumour size (*P* = 0.006) and operability (P = 0.015) and patient survival (Table 4; Figs. 3 and 4). There was no significant association between all treatment modalities and survival rate (*P* = 0.063) (Table 4; Fig. 5).

**Table 4:** Survival by characteristics and treatment of ATC

	Mean				Median1`				
			95% Confidence Interval				95% Confidence Interval		P*
	Estimate	SE	Lower Bound		Estimate	SE	Lower Bound	Upper Bound	
Grading									
A	7.42	2.17	3.17	11.68	7.00	1.30	4.43	9.56	
B	3.71	1.30	1.15	6.27	2.00	0.43	1.14	2.85	
C	5.50	2.64	0.32	10.67	2.00	1.83	0.00	5.60	0.441
Size									
≤ 4 cm	1.50	0.28	0.93	2.06	1.00				
> 4 cm	6.56	1.36	3.89	9.22	5.00	2.00	1.08	8.92	0.006
WBCs									
< 10,000	6.84	1.60	3.69	9.99	5.00	2.39	0.30	9.69	
≥ 10,000	3.14	1.20	0.78	5.50	2.00	0.39	1.21	2.78	0.104
Intervention									
Operable	7.41	1.73	4.00	10.82	7.00	2.30	2.47	11.52	
Non-operable	2.75	0.64	1.48	4.02	2.00	0.45	1.10	2.89	0.015
Type of therapy									
Chemotherapy	3.500	2.500	0.000	8.400	1.000				
Radio-therapy	2.000	0.000	2.000	2.000	2.000				
Surgery	3.250	1.797	0.000	6.772	1.000	2.000	0.000	4.920	
Chemo and radio	3.000	0.707	1.614	4.386	2.000				
Multiple modality	9.500	7.500	0.000	24.200	2.000				
Sympto-matic treatment	1.000	0.000	1.000	1.000	1.000				
Radio surgery therapy	9.500	2.187	5.213	13.787	8.000	1.225	5.600	10.400	0.063
Overall	5.550	1.178	3.242	7.858	3.000	1.491	0.078	5.922	

*By Log Rank (Mantel-Cox)

## Discussion

Anaplastic thyroid carcinoma is one of the fastest-growing tumours in humans, with local invasion and a high rate of distant metastasis. It has a poor prognosis, with a mean survival rate of 3–6 months after diagnosis [14, 15]. However, according to the data available, it is one of the uncommon malignancies of humanity. In the current study, the incidence of ATC was 3.15% of all cases of thyroid cancer, and the overall survival rate was 5.5 months.

Females are more prone to thyroid disease in general and cancers in particular. In our study, which retrospectively selected ATC patients, females comprised 63.6% of the sample (with a male-to-female ratio of 1:1.7), which agrees with the published data in different national contexts [16–18]. Most patients were over 60 years (63.6%), reflecting the association of age with the oncological process, including ATC [5]. This is related to the effect of tumour suppressor gene inhibition in older age, apparently as part of the natural ageing process [19].

According to published literature, local symptoms of ATC most commonly begin with a rapidly evolving central neck mass (77%), followed by noticeable dysphagia (40%), voice change or hoarseness (40%), and stridor (24%) [7, 8]. Our study showed similar results in the way in which most patients (81.8%) presented with compressive symptoms. The disease progression to surrounding tissues is one of the most important aspects of pathophysiology since it directly impacts the treatment and prognosis. In our observation stage, IVB + IVC was about 63.6%, and studies of rapid extension to surrounding tissues have reported that 80% of ATC patients have disease in both thyroid and nearby tissues at their initial presentation [1, 4–8, 20].

However, the survival duration of our cases with stage IVB + IVC was not so different from group A, who lived for 3–11 months, while those in Groups B and C lived for 2 weeks to 10 months (with a median of 2 months). Three months is commonly reported to be the median survival window [21]. The relatively shorter duration of survival in our study (2 months) could be explained by the delayed diagnosis and modality of treatments in the studied cases.

Participants’ tumour size was > 4 cm among the majority (77.3%) of participants. Previous studies reported tumours ranging between 3.6 and 10.5 cm, but only 68.9% of cases were reported to be larger than 4 cm [16, 22]. Even though larger tumour sizes are bad prognostic factors [22], those with larger tumours survived longer than those with smaller ones in the current study. This discrepancy could be explained by the role of large size in increasing patient awareness and motivation to seek treatment at an earlier stage, thereby increasing the scope of treatment and survival prospects.

One of the prognostic factors in ATC is the level of WBC. A patient with a WBC level of less than 10,000 /µL has a better prognosis regarding the duration of survival and may live 11.3 months, compared to patients with leucocytosis who average 3.6 months [23, 24]. In our study, the difference between the leucocytosis and the non-leucocytosis group was not statistically significant. However, there was a difference in the survival window (2 weeks to 9 months for non-leucocytosis and 2 weeks to 3 months for leucocytosis patients). Thus, the total body reaction in the form of leucocytosis may adversely affect the survival duration.

The incidence rate of thyroid cancer is increased, despite advances in early detection and understanding of the disease. Advanced molecular techniques such as DNA zyme are needed to diagnose accurately. Additionally, they may substantially impact the prognosis categorisation of thyroid cancer and provide a more accurate diagnosis [25, 26]. However, our area still uses traditional methods such as clinical examinations, x-rays, FNAC, and histopathological examinations.

The FNAC as a diagnostic tool in ATC has good support for its range of accuracy: 89.71% accuracy in diagnosis for 94.7% of ATC cases diagnosed by FNAC [27]. In our study, FNAC was used for all patients, and it was accurate in 20 (90.9%) cases as a diagnostic tool, given that all our cases were included and based on histopathological reports that assured the accuracy of FNAC for such malignancy.

Recent studies showed the diagnosis of ATC is difficult due to mimic to other carcinomas in the neck like poorly differentiated thyroid cancer, spindle cell variant of medullary carcinoma, solitary fibrous tumor and spindle epithelial tumor with thymus-like differentiation, leiomyosarcoma of the thyroid gland and different pathological changes that may affect normal ectopic thyroid [28, 29]. In addition to the above factors, the mentality and absence of a central hospital for the management of oncological cases in our region can be regarded as another two factors that may affect the late presentation of cases, missing cases during the diagnosis process, and improper management.

Surgery is one of the best options for patients to remove the tumour completely or improve its local effects, like airway compression. However, in small, non-extended tumours, it could be considered a curative for a short duration, with potential application as a palliative measure in inoperable patients. However, it could potentially extend survival outcomes in the case of surgery combined with chemoradiation [22]. This study found that surgery showed a better median survival time of 7 months for operated cases, compared to 2 months for non-operable ones. The 9% of patients who lived more than one year (a remarkable duration for ATC patients) were those who had undergone surgery as a part of treatment (in addition to chemoradiotherapy).

The main treatment modality in the group of patients with local and distant metastasis was adjuvant therapy, with or without debulking. Trials for different chemotherapy variants do not significantly change the duration of survival in ATC [30]. However, hypo fractionated radiotherapy significantly prolonged the median survival duration in this category of patients [31].

Single modality treatment does not show good prognostic effects; although surgery was one of the mainstays of treatment in itself (without additional treatment), it only achieved a 2-month survival rate. Similarly, when used alone, radiotherapy and chemotherapy achieved 2 and 3.5 months of survival, respectively. Conversely, multimodal treatment extended the survival window to 9.5 months – in particular, this was achieved by surgery in combination with radiotherapy (combined without chemotherapy). Combining chemotherapy and radiotherapy without surgery only produced a mean survival time of 3 months.

In addition, treatment approaches based on molecular pan-inhibitors of Aurora kinases, such as VX-680/MK-0457, SNS-314, and ZM447439 on human ATC-derived cell lines and urokinase-type plasminogen activator, have been developed [32, 33]. However, the clinical trials of these agents do not yet consider them in the management of ATC in our region.

## Conclusion

ATC and its treatment in the Kurdistan Region exhibit the same tendencies as elsewhere in the world. This study shows the superiority of early diagnosis and multimodality treatment as two important factors that may provide better survival rates. However, this study is limited by its small number of cases. The retrospective nature of this study limits the approach to many specific issues of concern for such pathology. At the same time, local specifications like the inaccessibility of radiotherapy and chemotherapy shortly after surgery, and the lack of proper awareness and screening programs in the country, make the diagnosis and prognosis of this pathology much worse than they could be. ATC is a rapidly growing cancer that, fortunately, is rare. Early diagnosis and multimodality treatment may provide a better quality of life and survival time for patients, and awareness of this among healthcare decision makers in the Kurdistan Region should be encouraged to increase screening and healthcare-seeking behaviour among the public.

## Electronic supplementary material

Below is the link to the electronic supplementary material.

Supplementary Material 1: Information Sheet

Supplementary Material 2: Ethics committee approval sheet

## Data Availability

The datasets used and/or analysed during the current study are available from the corresponding author upon reasonable request.

## Abbreviations

**COVID-19**: Coronavirus disease
**ATC**: Anaplastic thyroid carcinoma
**FNAC**: Fine needle aspiration cytology
**AJCC**: American joint committee on cancer
**CT**: Computed tomography
**FDG**: Fluorodeoxyglucose
**PET**: Positron emission tomography
**MRI**: Magnetic resonance imaging
**SD**: Standard deviations
**NGS**: Next generation sequencing
**CRISPR**: Clustered regularly interspaced short palindromic repeats
