# Supplementary material for: Evaluation of anaplastic thyroid carcinoma in the Kurdistan region of Iraq
Source: BMC Surg. 2022 Oct 21;22:364. doi: 10.1186/s12893-022-01810-w (PMC9587643; doi:10.1186/s12893-022-01810-w)
Supplement: Supplementary file 1 — Supplementary Material 1: Information Sheet [file 12893_2022_1810_MOESM1_ESM.docx]

**Title: -**, Evaluation of anaplastic thyroid cancer in Kurdistan region of Iraq

**Part I: Information Sheet**

**Introduction**

We are a group of specialist general surgeons, working in Erbil City hospitals, Were

researching Evaluation of anaplastic thyroid cancer in Kurdistan region of Iraq. We will provide you with information about the procedure, you don't have to decide today whether or not you should participate in this research. Before you decide, you can talk to anyone you feel comfortable with about the research. This consent form may contain words that you do not understand. Please ask me to stop as we go through the information and I will take the time to explain. If you have questions later, you can ask them of me or another researcher.

**Purpose of the research**

The purpose of this study retrospectively was to analyze clinical and pathological features of ATC in order to assess treatment procedures and its outcome.

**Type of Research Iobservation**

In this retrospective study, 700 cases of thyroid cancer were examined, out of which 22 were included as cases of ATC. The data were obtained from various hospitals and health care centers (Rizgary, Nanakaky, Hewa and Azadi Teaching Hospitals with their oncologic units) throughout Kurdistan region (Erbil, Sulaymaniyah and Duhok) cities in north of Iraq. All 22 cases were pathologically diagnosed as ATC depending on FNAC and excisional biopsy. Demographic characteristics of patients (age, sex, place of residence and life on endemic goiter); clinical presentation, diagnostic procedures, therapeutic approaches, their outcomes and Survival of ATC patients were collected and registered using a questionnaire, Additional data has been collected from patients’ relatives by direct interviewing or calling.

**Participant Selection**

You were selected as you need to have surgical removal of thyroid gland. The most serious complications are hypocalcemia, recurrent laryngeal nerve injury, incomplete removal of tumor and bleeding .

**Voluntary Participation**

Your participation in this research is entirely voluntary. It is your choice whether to participate or

not. If you choose not to participate all the services you receive at this Centre will continue and

nothing will change.

**Procedures**

After your approval, you will undergo surgery to remove the thyroid. This research will add a medication that reduces the infection rate.

For patients under 18 years old the approval is taken from the parents.

**Duration**

The research will be conducted over four years, follow up to know the prognosis and survival rate.

**Risks**

This includes the risk of general anesthesia and surgical procedure which include bleeding, sepsis, and nerve injury.

**Benefits**

The anticipated benefits may include life-saving measures, increasing survival rate and way of therapy.

The study group will afford for cost of surgery

**Confidentiality**

All patient's information will be confidential unless the patient chooses to disclose

information to a third party.

**Sharing the Results**

The data will be analyzed without attributing to names and personal information. The results will

be published later in a scientific journal also without revealing personal information or patients'

names.

**Right to Refuse or Withdraw**

You do not have to take part in this research if you do not wish to do so, and choosing to

participate will not affect your life in any way. You may stop participating in the trial at any time

that you wish.

**Who to Contact?**

If you have any questions, you can ask them now or later. If you wish to ask questions later, you

may contact any of the following:

| Title: Assistant lecturer | Name: Dilshad Hamad Mustafa |
| --- | --- |
| Qualification: MSc | Affiliation: HAWLER MEDICAL |
| Phone: +9647504482142 | Email: Dilshad@hmu.edu.krd |

| Title: Assist.proff | Name: Baderkhan Saeed Ahmed |
| --- | --- |
| Qualification: FI.B.M.S. | Affiliation: HAWLER MEDICAL |
| Phone: +9647504491528 | Email: baderkhan.saeed@hmu.edu.krd |

| Title: Title: Assist.proff | Name: Rawand Musheer Haweizy |
| --- | --- |
| Qualification: FI.B.M.S. | Affiliation: HAWLER MEDICAL |
| Phone: +9647504547694 | Email: Rawand.haweizy@hmu.edu.krd |

| Title: Lecturer | Name: Azhy Muhammed Dewana |
| --- | --- |
| Qualification: FI.B.M.S. | Affiliation: HAWLER MEDICAL |
| Phone: +9647504482305 | Email: azy.rwandizy@hmu.edu.krd |

| Title: Assist.proff | Name: Rawand Musheer Haweizy |
| --- | --- |
| Qualification: FI.B.M.S. | Affiliation: HAWLER MEDICAL |
| Phone: +9647504464548 | Email: Rawand.haweizy@hmu.edu.krd |

This proposal has been reviewed and approved by [Hawler Medical University], which is a

committee whose task is to make sure that research participants are protected from harm.

**Part II: Certificate of Consent**

**I have read the foregoing information, or it has been read to me. I have had the opportunity to ask questions about it and any questions I have been asked have been answered to my satisfaction. I consent voluntarily to be a participant in this study**

Print Name of Participant………………………………………………

Signature of Participant………………………………………………...

Date…………………………………….

Day/month/year

**If illiterate 1**

**I have witnessed the accurate reading of the consent form to the potential participant, and**

**the individual has had the opportunity to ask questions. I confirm that the individual has**

**given consent freely.**

**Print name of witness…………………………………. Thumbprint of participant**

**Signature of witness……………………………………**

**Date………………...**

**Day/month/year**

**Statement by the researcher/person taking consent**

I have accurately read out the information sheet to the potential participant, and to the best of my ability made sure that the participant understands that the following will be done:

1.

2.

3.

**I confirm that the participant was allowed to ask questions about the study, and all the questions asked by the participant have been answered correctly and to the best of my ability. I confirm that the individual has not been coerced into giving consent, and the consent has been given freely and voluntarily.**

**A copy of this ICF has been provided to the participant.**

**Print Name of Researcher/person taking the consent………………………….**

**Signature of Researcher /person taking the consent…………………………**

**Date…………………………**

**Day/month/year**

**_________________**

*^1^* A literate witness must sign (if possible, this person should be selected by the participant and should have no

connection to the research team). Illiterate that participants should include their thumbprints as well.
