# Supplementary figures and images for: Evaluation of anaplastic thyroid carcinoma in the Kurdistan region of Iraq
Source: BMC Surg. 2022 Oct 21;22:364. doi: 10.1186/s12893-022-01810-w (PMC9587643; doi:10.1186/s12893-022-01810-w)

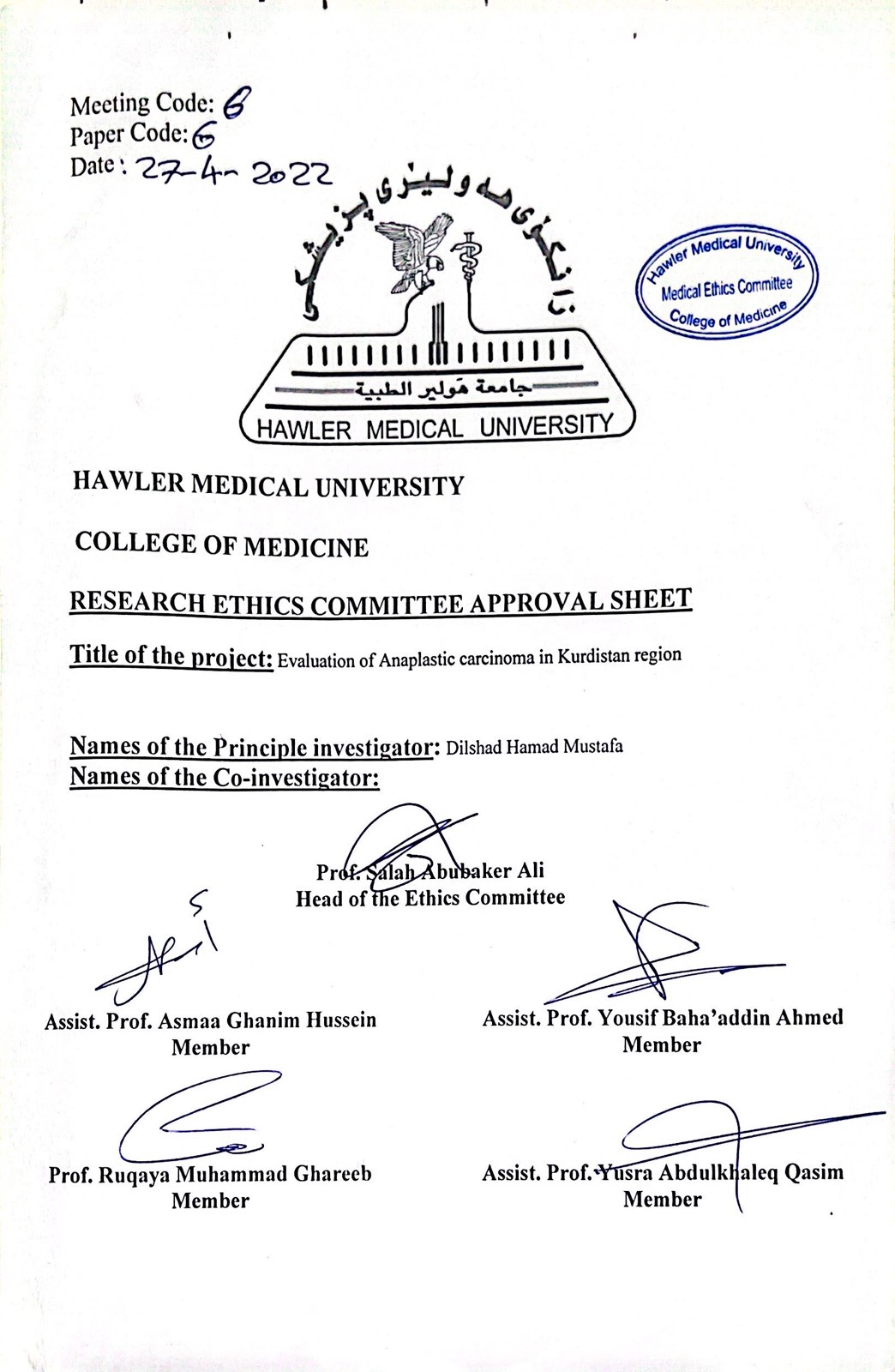

Supplement: Supplementary file 2 — Supplementary Material 2: Ethics committee approval sheet [file 12893_2022_1810_MOESM2_ESM.jpg]
